# Emerging Technologies and Methods for Musculoskeletal Tissue Repair and Regeneration

**DOI:** 10.1155/2018/8953079

**Published:** 2018-10-04

**Authors:** Cho-Pei Jiang, Liping Wang, Weijie Fu

**Affiliations:** ^1^National Formosa University, Taiwan; ^2^University of South Australia, Adelaide, Australia; ^3^Sport & Harvard Medical School, Shanghai University of Sport, China

As professionals working in the field of musculoskeletal tissue repair and regeneration, we know that bioprinting and novel medical design become the key tool to produce the implantable scaffold, mimic bone, and surgical guidance device for injury site. In addition, potential molecular signaling and tissue engineering of musculoskeletal damage and degeneration, the developments and improvements in biomechanical theory, scaffold fabrication methodology, and emerging technique also need to be focused on for orthopedic surgery and rehabilitation.

In this special issue, a 3D printer was used to make bone regeneration membrane and a photo-curable resin was introduced to make bone graft. A 3D-printed Titanium alloy plate was also treated for acetabular facture. Two studies related to osteocyte and osteoblast discussed the effect on bone regeneration. miR-142-5p in bone marrow-derived mesenchymal stem cells was verified to promote osteoporosis. Furthermore, two articles used computational method to simulate the effect of muscle direction on human lumbar spine and biomechanical responses of the osteocytes to the compressive stimulus. For clinical area, a role for postoperative negative pressure wound therapy in multi-tissue hand injuries was reported and methods for skeletal muscle tissue repair and regeneration were also reviewed.

Novel medical devices, new biomaterial for musculoskeletal tissue repair, and the clinical result of bone regeneration are expected to be developed in the immediate or near future. 3D printing and biomechanical analysis will be combined to predict or improve the current treatment method in clinical research and finally valuable assessments.

